# Awake versus Asleep Deep Brain Stimulation Surgery: Technical Considerations and Critical Review of the Literature

**DOI:** 10.3390/brainsci8010017

**Published:** 2018-01-19

**Authors:** Ryan B. Kochanski, Sepehr Sani

**Affiliations:** Department of Neurosurgery, Rush University Medical Center, Chicago, IL 60612, USA; Ryan_B_Kochanski@rush.edu

**Keywords:** awake, asleep, DBS, targeting, stereotaxis

## Abstract

Advancements in neuroimaging have led to a trend toward direct, image-based targeting under general anesthesia without the use of microelectrode recording (MER) or intraoperative test stimulation, also referred to as “asleep” deep brain stimulation (DBS) surgery. Asleep DBS, utilizing imaging in the form of intraoperative computed tomography (iCT) or magnetic resonance imaging (iMRI), has demonstrated reliable targeting accuracy of DBS leads implanted within the globus pallidus and subthalamic nucleus while also improving clinical outcomes in patients with Parkinson’s disease. In lieu, of randomized control trials, retrospective comparisons between asleep and awake DBS with MER have shown similar short-term efficacy with the potential for decreased complications in asleep cohorts. In lieu of long-term outcome data, awake DBS using MER must demonstrate more durable outcomes with fewer stimulation-induced side effects and lead revisions in order for its use to remain justifiable; although patient-specific factors may also be used to guide the decision regarding which technique may be most appropriate and tolerable to the patient.

## 1. Introduction

Deep brain stimulation (DBS) surgery is an effective treatment for movement disorders such as essential tremor (ET), dystonia and Parkinson’s disease (PD) [1,2,3]. Despite these established benefits, a tremendous degree of heterogeneity exists in the way DBS surgery is performed across centers [4]. Traditionally, surgery has been performed “awake” with utilization of microelectrode recording (MER) and/or intraoperative test stimulation; however, it is suggested that increased risk of intracranial hemorrhage (ICH) and cognitive decline may be related to the use of MER [5,6,7]. There is also no consensus on whether MER adds value or benefit to DBS surgery by improving outcomes when compared to DBS under general anesthesia without neurophysiological recording or stimulation, also referred to as “asleep” DBS surgery [8,9]. Because of logistical concerns, no randomized trials comparing outcomes of asleep and awake DBS surgery have been or are likely to be performed [10,11]. Therefore, comparisons can be made only with, at best, class II evidence through analysis of retrospective data [11]. This review will highlight the factors that have led to the trend toward asleep DBS while also providing technical comparisons between asleep and awake DBS with MER. 

## 2. Advances in Neuroimaging: Changing the Paradigm

Classically, the purpose of performing awake surgery with MER has been to map the borders of deep brain nuclei that were not easily or well visualized on conventional imaging sequences [12,13,14,15,16]. Because of suboptimal visualization of these structures, reliance was placed on stereotactic atlases that attempted to “standardize” the x, y, z coordinates of these structures with respect to the anterior commissure-posterior commissure AC-PC plane. Studies have demonstrated significant heterogeneity between patients in regard to the size, orientation and coordinates of deep brain nuclei [12,17,18,19]. Moreover, it has been demonstrated that coordinate-based lead location does not correlate with outcomes in patients with PD [20]. As the resolution of imaging modalities such as magnetic resonance imaging (MRI) has improved, many of the structures targeted during DBS surgery can be directly visualized on preoperative imaging and subsequently targeted [21,22]. This notion has also been supported by the concordance of MER with stereotactic position of the microelectrode within the intended structure, thus potentially obviating the need for awake surgery with MER and intraoperative stimulation [13,21,23]. To date, no consensus exists regarding optimal imaging sequences used for direct targeting of deep brain structures like the subthalamic nucleus (STN), with significant heterogeneity between centers [24]. Both T2 and susceptibility-weighted imaging (SWI) sequences have been shown to correlate well with the neurophysiological target [12,13]. The use of Fast Gray Matter Acquisition T1 Inversion Recovery (FGATIR) has also shown utility in visualization of structures like the STN, internal/external globus pallidus (GPi/GPe), and substantia nigra (SNr) [25]. More recently, quantitative susceptibility mapping (QSM) has shown promise in direct targeting of the STN [12,23,26]. Diffusion tensor imaging (DTI) may also provide utility in asleep DBS by defining boundaries of targets in relation to critical white matter tracts, as demonstrated by concordance between electrophysiological tissue activation and the distance between visualized corticospinal tract fibers [27]. 

Asleep DBS can therefore be performed on any deep brain structure that can be visualized radiographically. The majority of literature on asleep DBS pertains to direct targeting of both the STN and GPi, as both can be directly visualized on both 1.5 and 3T MRI using the sequences described above. To date, asleep lead implantation into the ventral intermediate nucleus of the thalamus (VIM), which cannot be directly visualized on conventional MRI sequences, has been performed with promising short and intermediate term results; although targeting is still performed using conventional atlas coordinates [28]. Tractography-based identification of the dentato-rubro-thalamic tract and adjacent corticospinal tract holds significant promise in improving direct targeting with side-effect avoidance [29,30]. Some centers have therefore shifted entirely away from awake DBS with MER, whereas others rely on factors such as patient preference and target selection to help dictate whether to perform the surgery asleep or awake [31,32,33]. 

Asleep DBS, depending on the center, has generally been performed in either one of two ways: intraoperative CT (iCT) confirmation or intraoperative MRI guidance (iMRI) [10,28,34,35,36,37,38]. These two techniques not only differ in the imaging modality utilized but also in the manner by which the imaging is used. Typically, iCT is used as a confirmatory technique, whereby an iCT is performed after the DBS lead has been placed at the predetermined stereotactic target. The iCT images are then merged to the preoperative MRI and the stereotactic position of the lead is determined based upon the CT-MRI fusion [10,34]. This confirmation allows for verification that the lead has been placed within an acceptable error to the radiographic target but does not allow for real-time changes in trajectory prior to lead placement. This is in contrast to iMRI techniques whereby an iMRI is performed either prior to or following dural opening and before placement of the lead at target [35,36]. The trajectory of the lead is extrapolated down to target using the software and adjustments can be made using the skull-mounted head stage in order to adjust for errors in targeting. Therefore, MRI can be used to actually guide the placement of the lead in real-time. Although we do not perform asleep DBS at our center, we have developed a technique that uses iCT guidance to adjust for any trajectory errors of the microelectrode prior to dural opening and advancement to target [39,40,41]. Theoretically, this technique could be used during asleep DBS using iCT in a similar manner as iMRI guidance to improve stereotactic accuracy, with the exception that iMRI allows for “live” target adjustments which can accommodate for brain shift. 

## 3. Targeting Accuracy

Asleep DBS is performed under the theoretical assumption that stereotactic accuracy in relation to the specified target leads to positive outcomes that are not inferior to awake DBS surgery using MER [32]. Therefore, stereotactic accuracy of the lead in relation to the planned target is crucial. Asleep DBS surgeries utilizing iMRI guidance have demonstrated a radial targeting error of 0.6–1.2 mm [35,42,43,44], while studies assessing accuracy of DBS lead placement using iCT have shown an error ranging from 0.8–1.24 mm [10,28,45]. It should be emphasized that asleep DBS surgery using confirmatory iCT is reliant upon the assumption of an accurate CT-MRI merge. While a number of studies have validated this methodology, there still may exist a small degree of error between the fusion of imaging modalities that may lead to suboptimal targeting [34,46,47]. In addition to lack of reliance on image fusion, another advantage of asleep DBS under iMRI guidance is the ability to account for brain shift following dural opening. Through analysis of iMRI studies, it has been previously shown that the degree and directionality of brain shift is often unpredictable, with shifting of deep brain structures by as much as 2 mm following dural opening [48]. 

During awake DBS surgery, the anatomic target is further refined by electrophysiological mapping. There is a paucity of published data on whether MER target refinement results in a significant deviation of DBS lead placement such that the deviation, on average, is greater than the inherent targeting error associated with DBS lead placement itself. Smith and Bakay calculated an error of 2.62 ± 1.5 mm between the intended MER-optimized target and final DBS electrode location as determined on post-operative MRI, and they hypothesized that this error may be a function of lead deviation into one of the prior, undesired MER tracks [49]. Stereotactic accuracy of the microelectrode itself is also critical, particularly when performing serial MER tracks directed toward a radiographic or atlas coordinate-derived target. There is also a paucity of data pertaining to stereotactic accuracy of the microelectrode in relation to the planned trajectory, and based on our experience, it cannot be assumed that the microelectrode will follow the exact same path. We have analyzed 227 MER trajectories (VIM, GPi, STN) following iCT, and found a radial error of 1.2 ± 0.2 mm compared to the intended target (unpublished data). These errors are likely related to mechanical imperfections within the frame, arc, guide tube, and headstage which can contribute to lead deviation in either awake or asleep DBS surgery [50].

## 4. Radiographic vs. Neurophysiological Target

The goal of both asleep and awake DBS with MER is to provide the highest degree of accuracy in relation to the radiographic and neurophysiological targets, respectively. Theoretically, these targets should be one and the same; however, several important factors can lead to errors in targeting therefore creating a discordance between the optimal radiographic and neurophysiological target. 

In our experience with awake STN DBS, we have encountered 25% (38/150) of hemispheres whereby the stereotactic targeting (by the microelectrode) was highly accurate (<1 mm radial deviation) to the desired target but MER and/or intraoperative stimulation was considered suboptimal for reasons including little or no electrophysiological recordings consistent with the target or unacceptably low side-effect threshold with stimulation. While such findings can likely be explained by brain shift, it is because of these instances that we feel MER continues to play an integral role in target confirmation during DBS surgery. While, iCT-MRI fusion techniques have been validated, even small merge errors combined with brain shift are likely the major factors leading to the discordance between optimal radiographic and neurophysiological targets [34,46,48,51,52,53]. Ivan et al. reported that the extent and directionality of brain shift can be unpredictable and lead to shifting of deep brain structures by as much as 2.1 mm [48]. Shifts of this magnitude can provide a false sense of electrode localization when iCT (after dural opening and brain shift) is merged with a preoperative CT or MRI, which may ultimately lead to a discordance between preoperative radiographic target and optimal neurophysiological target. These small errors induced by brain shift and iCT-MRI fusion can cause significant deviations of either the microelectrode or DBS electrode from its intended target. 

## 5. Outcomes

Despite the current body of literature demonstrating adequate accuracy and precision with asleep DBS surgery via direct targeting, the ultimate assessment is whether clinical outcomes, both short and long term, are comparable to awake DBS using MER [32]. Additionally, whether asleep DBS is safer, with a lower complication rate due to lack of MER passes and effect on the patients’ post-operative neuropsychological outcomes, remains largely unknown at this time. Due to a lack of single or multicenter randomized trials, the majority of outcome data on asleep DBS is based on retrospective comparison to prior outcome studies involving awake DBS surgery with MER [11]. Conclusions of these studies must be drawn with caution as comparisons are made between different surgeons performing the surgical procedure via a heterogeneity of techniques (i.e., frame/frameless, MER with intraoperative stimulation, intraoperative stimulation only, concurrent MER recording vs. serial MER, etc.). Nonetheless, multiple centers have published promising outcome data for asleep DBS implantation into the STN and GPi using either iCT or iMRI [31,42,43,44,45]. A recent meta-analysis by Ho et al. attempted to retrospectively compare 139 different patient cohorts that underwent awake surgery with MER to 16 cohorts undergoing asleep DBS [11]. Importantly, 7 of the asleep DBS cohort studies were still performed with MER under general anesthesia which certainly confounds the results. Nonetheless, they found no difference in lead placement radial error but a significant decrease in number of brain penetrations (1.4 vs. 2.1, *p* = 0.006) as well as percentage of intracranial hemorrhage (0.3 vs. 1.1%, *p* < 0.001) and infections (0.7 vs. 1.4%, *p* < 0.001) per lead in the asleep cohorts [11]. Furthermore, they found no significant difference between groups in percent change in unified Parkinson’s disease rating scale (UPDRS) II, III off medication, UPDRS III on medication, and levodopa equivalent dose (LEDD) scores at an average follow-up of 14.6 months in the awake and 11.6 months in the asleep cohort [11]. They also found no difference between operative time between cohorts; although this is likely a heterogeneous comparison as no distinction is made in regard to whether surgeries were performed as a single stage or in separate stages. Amongst the asleep cohorts, comparisons between imaging modality used (iCT vs. iMRI) could not be made because of inadequate numbers. 

Proponents of awake DBS with MER emphasize its ability to test for intraoperative side effects and therapeutic window of stimulation, which should theoretically lead to fewer postoperative side effects during programming as well as a longer effective duration of DBS efficacy. Ho et al. found a significant decrease in stimulation-induced side effects as measured by UPDRS IV scores in the ‘off’ medication state in the awake group in comparison to the asleep group [11]. Long-term ability to increase DBS settings with disease progression and whether awake DBS offers an advantage has not been evaluated. 

Several retrospective analyses have attempted to compare awake and asleep cohorts at a single center. Nakajima et al. retrospectively compared percentage of improvement in UPDRS III motor scores between 68 patients who underwent awake STN DBS lead implantation with 14 patients who underwent asleep DBS under general anesthesia and found no significant difference in improvement between cohorts at 12–14 months follow-up [54]. Another center retrospectively compared cohorts of 14 asleep MRI-guided STN DBS implantation patients with 23 awake MER-guided cases and found no significant differences in LEDD at 6 months follow-up [55]. More recently, Brodsky et al. demonstrated no significant difference in improvement of UPDRS III scores between a prospectively analyzed asleep cohort and retrospectively analyzed awake with MER cohort at 6 months in PD patients implanted into either the STN or GPi using iCT confirmation; however, they found significantly higher improvements in PDQ-39 summary index as well as cognition and communication subscores in the asleep cohort [31]. As most of the prior studies comparing awake and asleep cohorts have only assessed motor outcomes, further studies similar to Brodsky et al., but preferably including prospectively studied cohorts, would be of value in understanding the effects of awake and asleep DBS on factors such as speech and cognition. 

As previously mentioned, the general concept of asleep DBS is based upon targeting of deep brain nuclei that can be visualized on specific MRI sequences. Because the VIM nucleus of the thalamus cannot be visualized with the current clinically available MRI sequences, the general consensus among surgeons is to perform the implantation awake with MER and/or intraoperative test stimulation. Chen et al., retrospectively compared ET patients who underwent implantation into the VIM either awake with intraoperative test stimulation versus asleep without test stimulation and found no significant difference in the percentage of functional improvement at an average follow-up of 17 months [28]. Targeting was performed based upon a standardized set of x, y, z coordinates in relation to the AC-PC plane in all patients with only 1 of the total 17 asleep surgery patients requiring a second brain penetration because of a lead placement radial error of >2 mm. Interestingly, none of the 17 asleep patients required return to the operating room within 6 months for lead repositioning due to adverse stimulation effects despite targeting being based upon the same set of standardized coordinates for all patients. Furthermore, the final Euclidean error of the DBS lead was significantly higher in the awake group (1.7 mm vs. 1.2 mm, *p* = 0.01) [28]. These findings challenge the utility provided by MER/intraoperative stimulation during awake surgery. 

There is limited literature on the impact of complications associated with the use of general anesthesia during asleep DBS surgery. Although a recent study by Chen et al. found no difference in postoperative complications and 30-day readmission rates between retrospectively analyzed awake and asleep cohorts, they focused specifically on complications directly associated with the procedure (i.e., lead repositioning, infection, hemorrhage, altered mental status) and not on systemic complications such as venous thromboembolism (VTE) [56]. The surgical literature has demonstrated a direct correlation between time under general anesthesia and risk for VTE [57]. Although this has not been studied in asleep DBS surgery, one could surmise that the risk of complications such as VTE or pneumonia would be greater when performing the surgery under general anesthesia. 

As initially stated, the establishment of a randomized control trial comparing awake and asleep DBS cohorts presents significant challenges, and two prior randomized trials between awake and asleep DBS for both ET and PD resulted in premature closure at 6 months due to low recruitment rates [32]. Furthermore, a limited number of centers exist that can perform both awake and asleep surgery with equipoise. Although outcome data for asleep DBS appears promising, current follow-up periods in the asleep studies may not have yet captured patients who may require lead repositioning/revision [32]. With the reported rate of intracranial DBS lead revision already as high as 15.2–34%, longer-term outcome data between asleep and awake techniques would be of significant value in determining whether the use of MER should remain justifiable in DBS surgery [58]. It is also important to note that retrospective comparison studies between awake and asleep DBS have often compared asleep DBS using advanced intraoperative imaging to awake DBS with MER but without advanced intraoperative imaging. Contemporary awake DBS surgery using MER with utilization of intraoperative imaging and advanced imaging with target visualization is likely to have superior outcomes and thus would be of significant value in determining the true benefit of MER. As more centers trend toward asleep DBS with direct targeting, proponents of awake surgery with MER must demonstrate more durable outcomes with fewer stimulation-induced side effects/lead revisions over a long-term follow-up period in order to justify the added cost and complication risks associated with its use [8,9]. In regards to ideal intraoperative imaging modality used in asleep DBS surgery, further data is also needed to provide accurate comparisons in outcomes between iCT and iMRI. 

## 6. Future Directions

At present, the aforementioned studies provide evidence that asleep DBS techniques are highly accurate and confer at least equal efficacy in terms of outcomes in comparison to awake surgery. Importantly, DBS surgery in general should still be performed in a manner that the surgeon and center are most comfortable with, thus providing the patient with the best possible outcome. Hence, conclusions drawn through comparison of awake versus asleep techniques should be taken with caution as the more recent asleep surgery data has been reported from high-volume centers with significant experience in performing these cases, which like any new surgical technique, involves a learning curve. Centers that can perform both awake and asleep techniques with equipoise have attempted to characterize patient populations that might influence the decision to perform surgery in a certain manner [33]. Identification of factors such as claustrophobia, severe off-medication symptoms or a nonspecific fear of being awake during a surgical procedure, which can be addressed by performing asleep DBS, should theoretically increase the number of surgical candidates that might otherwise decline an awake procedure [33]. These are just a few of many factors that might preclude patients with either PD or ET from declining surgical intervention when considering DBS surgery when only given the option of undergoing awake surgery. If the surgeon feels that an equal or better outcome can be achieved with surgery done under general anesthesia, it may not only improve the patient experience, but also attract a greater number of patients electing to undergo DBS surgery. Henceforth, it is likely that in the future, both awake and asleep DBS will be part of the DBS surgeon’s armamentarium with a patient-specific tailored approach to implantation. 

While DBS surgery may be trending more towards an emphasis on stereotactic accuracy via direct image-based targeting, further improvements in DBS lead/device technology may also help mitigate complications that might arise from suboptimally placed DBS leads. With the introduction of leads capable of “steering” current in a specific radial direction, there may be a greater tolerability of DBS lead targeting error in comparison to the desired target [59]. Closed-loop DBS technology utilizing local field potential (LFP) analysis has also shown promise in reducing stimulation-induced dyskinesia in PD patients, while also providing more selective stimulation triggers/parameters [60,61,62,63,64]. LFP analysis has also shown promise in predicting optimal electrode implantation tracks in STN DBS surgery [65]. Although MER has provided invaluable neurophysiological information to help our understanding of basal ganglia neurophysiology, its utilization during DBS surgery will likely be determined on a multi-disciplinary basis based on patient- and center-specific factors. 
